# Draft Genome Sequence of Sphingobacterium sp. Strain HMA12, Which Encodes Endo-β-*N*-Acetylglucosaminidases and Can Specifically Hydrolyze Fucose-Containing Oligosaccharides

**DOI:** 10.1128/genomeA.01525-17

**Published:** 2018-02-22

**Authors:** Yibo Huang, Yujiro Higuchi, Kazuki Mori, Ryoko Yamashita, Nozomu Okino, Kosuke Tashiro, Kaoru Takegawa

**Affiliations:** a Department of Bioscience and Biotechnology, Faculty of Agriculture, Kyushu University, Hakozaki, Fukuoka, Japan

## Abstract

The genome sequence of the soil bacterium Sphingobacterium sp. strain HMA12, the culture supernatant of which exhibited endo-β-*N*-acetylglucosaminidase (ENGase) activity, was examined for ENGase-encoding genes. Here, we report the characterization of new genes of ENGases, obtained by whole-genome shotgun sequencing, that are capable of specifically hydrolyzing fucose-containing oligosaccharides.

## GENOME ANNOUNCEMENT

Endo-β-*N*-acetylglucosaminidase (ENGase) catalyzes the hydrolysis of the *N,N*′-diacetylchitobiose moiety of asparagine-linked oligosaccharides (1). Although many ENGases with versatile substrate specificity exist, no fucose-containing oligosaccharide-specific ENGases have been reported (2–4). To identify a fucose-containing oligosaccharide-specific ENGase, soil samples were screened, and a strain with ENGase activity in the culture supernatant was found. This strain was characterized by 16S rRNA gene analysis as a species of Sphingobacterium and was named strain HMA12. Whole-genome sequencing of Sphingobacterium sp. strain HMA12 was conducted to identify the genes encoding ENGases.

The draft sequence was generated by employing a whole-genome shotgun sequencing strategy using the MiSeq platform (Illumina, UK). A total of 4.7 Gbp was obtained from 1.31 × 10^7^ sequencing reads (727-fold coverage), with an average of 179 bp paired-end reads. These sequence reads were assembled using Platanus version 1.2.1, and 14 contigs were generated. The longest contig contained 3,197,939 bp, and the *N*_50_ size was 832 kb. Genome annotation was performed using Glimmer 3.02b, the BLAST 2.2.26 nonredundant protein sequence database, and InterProScan 4.8. The draft genome of Sphingobacterium sp. strain HMA12 was 6.47 Mbp, with an overall GC content of 40.7%, putatively encoding 5,770 genes. The mean and median gene lengths were 991 bp and 810 bp, respectively, and the gene density was 1,121 bp/gene.

Biochemical characterization of strain HMA12 involved examination of the sphingolipid content. The main components were ceramide phosphorylethanolamine and ceramide phosphorylinositol, both of which are known to be produced by Sphingobacterium spp. (data not shown; [5]). This result demonstrates that strain HMA12 indeed belongs to a Sphingobacterium species. The genome annotation demonstrated that Sphingobacterium sp. strain HMA12 contains many glycoside hydrolase (GH) family genes. A search for open reading frames (ORFs) showing high sequence similarities to known ENGase genes was performed, and five candidates were found. These were designated ORF1152, ORF1188, ORF2117, ORF3046, and ORF3750 and were located in contig01 (ORF1152, ORF1188, and ORF2117; GenBank accession number BEYR01000001), contig02 (ORF3046; accession number BEYR01000002), and contig03 (ORF3750; accession number BEYR01000003). Using Pfam (http://pfam.xfam.org/) and CAT (the Carbohydrate-active enzymes Analysis Toolkit), it was predicted that the five proteins belong to GH family 18, which normally exhibits ENGase activity. By using a variety of pyridylamino (PA)-oligosaccharides as the substrates, it was demonstrated that ORF1152, ORF1188, ORF3046, and ORF3750 recombinant proteins specifically hydrolyze fucose-containing biantennary oligosaccharides. In addition, these four ENGases hydrolyzed the *N*-linked glycans of rituximab (immunoglobulin G) as a glycoprotein containing fucosyl-sialobiantennary-type oligosaccharides. Homologs of Sphingobacterium sp. strain HMA12 ENGases were found to exist in a wide range of organisms, from bacteria to eukaryotes (6).

### Accession number(s).

The contig sequences of Sphingobacterium sp. strain HMA12 have been deposited in the DDBJ/EMBL/GenBank databases under the accession numbers BEYR01000001 to BEYR01000014.
